# Could sound be used as a strategy for reducing symptoms of perceived motion sickness?

**DOI:** 10.1186/1743-0003-5-35

**Published:** 2008-12-23

**Authors:** Joakim Dahlman, Anna Sjörs, Torbjörn Ledin, Torbjörn Falkmer

**Affiliations:** 1 Linköping University, Faculty of Health Sciences, IKE, Department of Rehabilitation Medicine, Linköping, Sweden; 2 Linköping University, Faculty of Health Sciences, IKE, Department of Otorhinolaryngology, Linköping, Sweden; 3 Jönköping University, School of Health Sciences, Jönköping, Sweden

## Abstract

**Background:**

Working while exposed to motions, physically and psychologically affects a person. Traditionally, motion sickness symptom reduction has implied use of medication, which can lead to detrimental effects on performance. Non-pharmaceutical strategies, in turn, often require cognitive and perceptual attention. Hence, for people working in high demand environments where it is impossible to reallocate focus of attention, other strategies are called upon. The aim of the study was to investigate possible impact of a mitigation strategy on perceived motion sickness and psychophysiological responses, based on an artificial sound horizon compared with a non-positioned sound source.

**Methods:**

Twenty-three healthy subjects were seated on a motion platform in an artificial sound horizon or in non-positioned sound, in random order with one week interval between the trials. Perceived motion sickness (Mal), maximum duration of exposure (ST), skin conductance, blood volume pulse, temperature, respiration rate, eye movements and heart rate were measured continuously throughout the trials.

**Results:**

Mal scores increased over time in both sound conditions, but the artificial sound horizon, applied as a mitigation strategy for perceived motion sickness, showed no significant effect on Mal scores or ST. The number of fixations increased with time in the non-positioned sound condition. Moreover, fixation time was longer in the non-positioned sound condition compared with sound horizon, indicating that the subjects used more time to fixate and, hence, assumingly made fewer saccades.

**Conclusion:**

A subliminally presented artificial sound horizon did not significantly affect perceived motion sickness, psychophysiological variables or the time the subjects endured the motion sickness triggering stimuli. The number of fixations and fixation times increased over time in the non-positioned sound condition.

## Background

In every environment in which people are exposed to motion sickness, either induced by visual or physical stimuli, the subject may get psychologically, as well as physically, affected [1-3]. The subject's susceptibility to motion sickness, in addition to previous experiences and anticipations related to the environment, determine the potential development of symptoms. Initial symptoms of motion sickness are highly individual, but typically include feelings of stomach awareness, increased salivation, yawning, dizziness and sweating [1,4]. Susceptibility to motion sickness can also be dependent of different medical conditions that facilitate the development of symptoms, both biologically and perceptually even during very subtle stimulation [2]. Subjects who have experienced motion sickness will bear witness to their specific initial symptoms of discomfort that often follow as a result of a subliminal increase in their sympathetic nervous system activity [5,6]. For most people exposed to motions, this initial sensation of increasing discomfort often initiates some mitigating strategy. However, for persons who are performing a demanding task or suffer from effects of medication or injury and at the same time being under the influence of motion sickness, performing deliberate mitigation strategies often fail. Furthermore, previous experiences of motion sickness related to a specific environment or condition often makes people attentive and more susceptible to motion sickness in that specific environment, or in similar environments [7], i.e. anticipations play a crucial role in the development of motion sickness. Hence, previous experiences contribute to how we react when exposed to motion [8,9]. However, if exposed to a specific environment repeatedly, adaptation usually occur after a few sessions depending on the duration of the exposure [10].

Considerable research has been devoted to identifying the specific autonomic responses associated with symptoms of motion sickness and many of them are measured together with ratings of perceived motion sickness [1,3,5,6,11,12]. By perceived motion sickness we refer to the state at which the symptoms of motion sickness are strong enough to be perceived by the subject. The development of motion sickness signs and symptoms are initially not observable or noticeable by the subject, which means that the autonomic responses to a motion sickness triggering stimulus starts before the subject is conscious of that he/she is affected by motion sickness. Through measurements of psychophysiological responses, the imbalance between the sympathetic and parasympathetic nervous system that normally occurs before the subject is aware of any change in wellbeing can be observed. The reason for using both subjective ratings and objective psychophysiological measurements for identifying and studying motion sickness in this study is to better assess the possible effects of a mitigating strategy, both on the conscious and unconscious level.

In many occupations in physically moving environments, limited possibilities for visual contact with the outside world are offered. These situations thus create good prerequisites for development of motion sickness symptoms; for instance onboard ships, airplanes and inside ground vehicles. Being under the influence of motion sickness in these types of environments affects performance and wellbeing, often to the point where interventions intended to stop symptoms have little or no effect [13]. Medication is, in most cases, of no use at that point in time [14]. The best prevention, or the most symptom reducing strategy, is to lie down as close to the centre of motion as possible and to reduce visual input. If possible, the affected person may also find relief in taking control of the motion by, for example, driving the car or steering the boat. Such control strategies require active handling, which in turn implies that the person has to leave his/her ordinary duties for some time [15]. As mentioned, one mitigation strategy used to treat motion sickness is to reduce the visual stimuli. In environments where simply closing the eyes is not an option, an alternative could be to reduce the stimuli input by reducing the number of fixated objects. This strategy also reduces the number of saccades, which further lowers yet another motion sickness triggering factor [16,17]. Previous research in this area has shown that motion sickness seems to be perceived both with foveally and peripherally presented stimuli, the latter giving rise to more vection than the former[18].

Previous research has supported the idea that by providing a reference to the outside world in a sealed off moving environment, the occurrence of motion sickness can be postponed, or in some cases even be reduced to a minimum [19,20]. Rolnick and Bles, [21] found that the perception of motion sickness in sealed off environments may be reduced or kept stable when subjects are provided a visually projected horizontal reference. Another study presented an Independent Visual Background (IVB) to a number of subjects in a driving simulator that was in accordance with the vestibular and visual information perceived [22]. This visual-based artificial horizon reduced perceptual errors and also reduced balance disturbance when presented in low frequencies of motion. However, simply picking one reference stimulus without paying further attention to the exact way this reference stimulus is presented may prove dubious, since presenting different reference stimuli in the same perceptual mode could yield quite disparate outcomes [22].

Very few studies have used other means of reference support than visually presented information for possible reduction of postural instability, vection and symptoms of motion sickness. For example, Petersen, Magnusson, Johansson, Åkesson & Fransson. [23] used sound as a cue in order to facilitate maintenance of postural stability and to provide a reference that would replace visual information. One condition provided the subject with a pitch tone that increased in intensity when the subject leaned forward and decreased when leaning backwards. Another condition provided the subject with audio pulses that gave the subject directional support in the horizontal plane. The results indicated that when given any constant audio feedback signal, body sway was significantly reduced. The results illustrate the potential importance of auditory cues for spatial orientation. Dozza, Chiari, Chan, Rocchi, Horak and Capello [24], used audio biofeedback (ABF) through a portable ABF-system to support subject upright stance and postural stability. The ABF provided an audio feedback signal when the subject leaned over, or lost upright stance. The audio signal was converged through a pair of headphones and the subjects were blindfolded, standing on a thick foam plate. Results indicate that audio biofeedback significantly reduces body sway in healthy subjects and can be used to treat postural instability by helping the brain to maintain posture.

Sound as a preventive countermeasure for motion sickness and other vertigo related conditions has rarely received attention. Previously mentioned research has used sound presented either in mono or stereo. To our knowledge, no studies using sound sources that indicate a specific position in the environment, to support the subject's perception, have been carried out. Positioned sound sources are commonly associated with 3D-sound and can be experienced when attending any modern cinema. The benefit of using positioned sound sources as a mitigating strategy for motion sickness is that it could affect the subject subliminally and thereby not require any additional cognitive attention. It is, however, important to keep the stimulation as subtle as possible, in order not to add further conflicting cues, or to enhance already experienced symptoms. It is unlikely to assume that positioned sound sources could eliminate symptoms completely, but a tentative hypothesis is that it could postpone the onset of perceived motion sickness or at least keep the symptoms at a constant lower level for some time, compared to a control condition. Hence, by supporting the subjects with an outside reference on a subliminal level, tasks that do require devoted attention can continue.

## Aim

The aim of the study was to investigate possible impact of a mitigation strategy on perceived motion sickness and psychophysiological responses, based on an artificial sound horizon compared with a non-positioned sound source.

## Methods

The study had a within-group design and was performed in a controlled laboratory setting.

### Subjects

The subjects were recruited through public advertisements. Applicants with perceived high susceptibility to motion sickness symptoms were of special interest and therefore selected for participation. Individuals with vestibular and or hearing dysfunctions, or who were on medication that could confound the psychophysiological measurements and/or contribute to nausea were excluded. A total of 6 men and 17 women, (mean age 29.0 years, range 20–51 years) volunteered to take part in the study. Written consent was obtained from the subjects after informing them of the possibility of acquired discomfort from exposure to motion stimulation. They were also informed of the right to withdraw from the experiment at any time without stating a reason. The subjects completed a screening questionnaire concerning their previous experiences of, and susceptibility to, motion sickness. The screening questionnaire was administered to explain possible outcome of the motion exposure and to create a better basis for the symptoms susceptibility among the subjects and was created by the authors based on experiences from previous studies. The subjects were asked to refrain from intake of anti-motion sickness medications and antihistamines 24 hours prior to the experiment.

The study was approved by the local Ethics Committee.

### Motion sickness-induction

**Figure 1:**
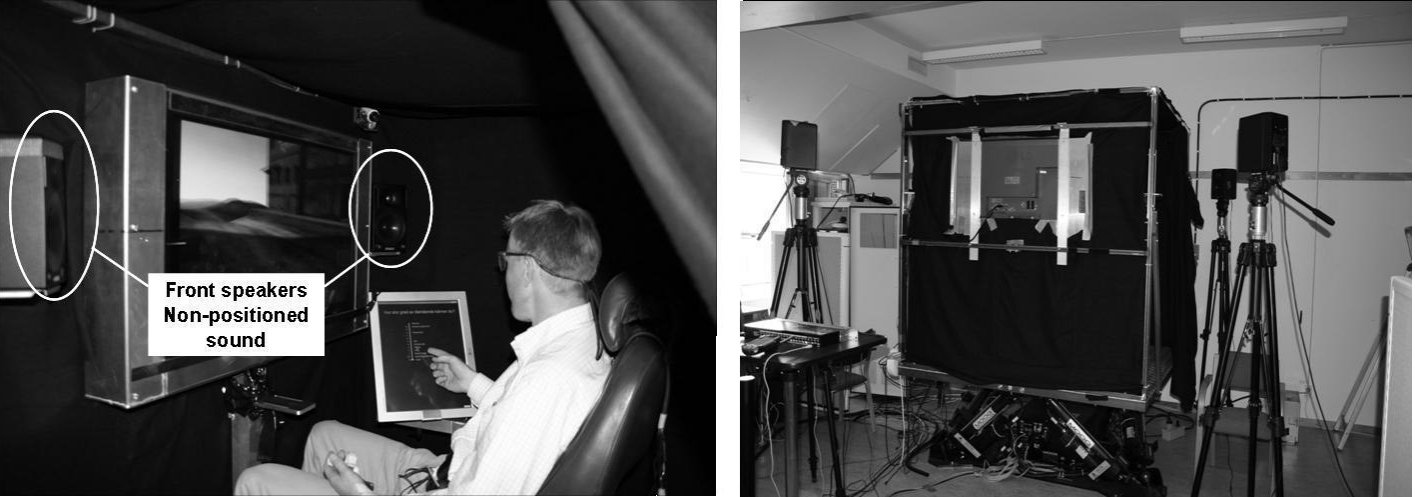
**Picture shows front speakers location inside cabin in the non positioned sound condition (left), speakers behind the subject not visible in picture**. Speakers positioned outside the cabin in the sound horizon condition (right). The right most screen displayed the Mal score questionnaire.

A motion platform (Moog 6dof2000E), shown in Figure 1, with six degrees of freedom producing low frequency movements similar to those of a sea vessel was used to provoke symptoms of motion sickness. The motion was a combination of roll, pitch and heave, each with maximum amplitude 0.1 m. To create a motion profile that would feel like random movements, three sine functions with frequencies 0.12 Hz, 0.15 Hz and 0.19 Hz were added to create the motion patterns. Different values for roll, pitch and heave were then obtained by phase-shifting the motion pattern. The subjects were seated in a chair located within a closed cabin on the platform to ensure that no outer points of reference were visible. A visual distraction task (a video showing a bird's view flying through a virtual terrain) was utilized to keep the subjects occupied and to refrain them from taking deliberate countermeasures against the development of motion sickness. The distraction task was to search for specific objects in the video. However, no performance measures were obtained from this task. The subjects were instructed not to make any head movements other than those necessary for administering the electronic questionnaire, which was located on a separate screen just beside the screen on which the secondary task was presented. None of the two screens used, or any other equipment in the enclosed area around the subject could provide any visual reference to the environment outside the platform.

### Sound

The sound used in this study was a so-called "pink noise"; i.e. low pass filtered white noise, with equal energy per octave [25] provided by four loudspeakers positioned in a square, either on or outside the platform, as shown in figure 1.

The subjects were tested in two conditions. In the first, hereafter called the "non-positioned sound" condition, the speakers were placed on the platform, whereas as in the second condition, hereafter called the "sound horizon" condition, the speakers were placed at the subjects' horizontal ear level outside the platform to create a fixed auditory reference.

The sound was equally loud in both conditions. For each speaker, the sound level was kept within 56–57 dB measured in the subject's seat. Sound level measurements were performed using Lvie IE-33J decibel meter and a HP IPAQ 5450. The microphone was placed at the subject's ears striving to measure the actual sound level experienced during the trials. The background sound level with the speakers turned off was 53 dB, which resulted in total sound level of 59.6–60.0 dB.

### Procedure

All subjects were given two separate trials, one in the non-positioned sound and one in the sound horizon, with a minimum of one week apart. They were not informed about which experimental condition they were exposed to until after they had performed both trials, and the debriefing session took place, which is further described below. On arrival, the subjects were given a chance to familiarize with the equipment and to ask questions. Each participant was instructed in advance to ride as long as he or she could, short of vomiting. Maximum duration of exposure for each trial was 40 minutes.

The subjects were exposed to the following experimental conditions during both trials:

#### (1) Five-minute rest period

Subjects were asked to rest comfortably on board the platform in front of a blank screen. The first half of these five minutes served as familiarization phase, whereas the last 2.5 minutes served as baseline. The subjects then completed the first subjective rating of perceived motion sickness using the electronic questionnaire.

#### (2) Motion sickness stimulation

The motion platform and video were initialized and continued running throughout the trial. Ratings of perceived motion sickness were obtained at 2 minute intervals using the electronic questionnaire, which took approximately 30 seconds to complete (i.e., an approximate cycle time of 2.5 minutes). While completing the questionnaire, the subject had to move his/her head slightly to the right, as seen in figure 1. During the trial, subjects were subjected to either the non-positioned sound or the sound horizon. Each subject performed one trial in each auditory setting in a randomly selected order, which meant that half of the group started with the non-positioned sound and the rest of the subjects with the sound horizon. The subjects were not informed about the order of the trials, or about the different sounds. The trial was terminated when either: a) the subject requested termination or b) the maximum duration of the trial had been achieved.

#### (3) Debriefing

After completing the first of the two trials, all subjects were given the chance to terminate the study before scheduling the second appointment. When both trials were completed, the subjects were fully informed about the purpose of the study in accordance with the approved ethical application.

### Measurements

#### Perceived motion sickness

An electronic questionnaire was constructed to measure subjective reactions to the experimental conditions and comprised question 1–10 of the Graybiel scale [26]. In total, the Graybiel scale consists of twelve questions concerning the severity of various symptoms of perceived motion sickness, e.g. pallor, nausea, dizziness, stomach awareness. A single global *malaise score *(Mal) ranging from 0–62 can be derived using a complex scoring and weighting system, further described by Miller & Graybiel [27]. The scale was presented to the subject on a touch screen on the platform with 2 minute intervals between each questionnaire. The touch screen could not be used as a visual reference to the outside environment or in any other way help the subject.

#### Psychophysiological responses

Measurements of heart rate (HR), skin conductance level (SCL), blood volume pulse (BVP), skin temperature (TEMP) and respiration rate (RR) were made using the MobileMe recording system (Biosentient Inc.). HR was computed beat-to-beat from electrocardiogram (ECG) recordings, which were measured via a standard lead II configuration. ECG recordings were made with a sample rate of 256 Hz. The electrodes used were disposable pre-gelled Ag/AgCl electrodes. SCL measurements were derived from disposable pre-gelled Ag/AgCl electrodes placed on the medial phalanges of the index and middle fingers of the left hand. BVP is a relative measure of vasomotor activity derived from a photoplethysmograph (PPG) transducer placed on the left ring finger. BVP was measured as changes in the peak-to-peak amplitude of the PPG signal in arbitrary units. TEMP recordings were derived from a thermistor placed on the little finger of the left hand. RR was computed breath-to-breath from the respiration (RESP) signal which was recorded using a strain gauge strapped around the chest. For SCL, PPG, TEMP and RESP signals, the sample rate was 32 Hz. All psychophysiological measurements were averaged over 2.5 minute intervals for the statistical analyses. All psychophysiological measurements were recorded throughout the entire exposure and baseline period.

*Eye movements *were recorded using a head mounted ViewPoint eye tracker [28], which recorded pupil size and the x and y position co-ordinates of the eye in 50 Hz. The coordinates were initially run through a centroid mode fixation generation analysis [29] in order to filter out fixations from other eye movement data. After that, the average number of fixations (NoFix) and fixation duration (Fixdur) over the time periods was calculated for each subject. In order to compare actual fixation time (Fixtime) across the conditions, NoFix was multiplied by Fixdur. The eye tracker was mounted on the subject's head and calibrated outside the platform using a standardized 16 dot grid [28].

#### Duration of exposure

The maximum duration i.e. Stop Time (ST) was measured as the time in minutes the subjects remained in each trial.

### Statistical analyses

Analyses were conducted using SPSS (version 15.0 for Windows). Motion sickness ratings, i.e. Mal, psychophysiological measurements (HR, SCL, BVP, RR and Temp), and eye movements (NoFix, Fixdur, Fixtime) were compared across conditions using paired samples t-tests. For Mal, HR, SCL, BVP, RR and Temp, the slope from baseline to termination was calculated for each subject, i.e., (Last measurement – baseline)/ST. For eye movement data, the slope was instead calculated from the first 2.5 min interval to termination since there were no baseline measurements. A positive slope, hence, indicates an increase over time and the larger the slope, the faster the increase. Paired samples t-tests were also used to investigate any differences in duration of exposure, i.e. ST between the two sound conditions and between the first and second trial. Pearson correlations were calculated to investigate relations between variables with Bonferroni correction applied for multiple testing [30]. Variables were tested for normal distribution with the Kolmogorov-Smirnov test for normality and variables not normally distributed were analyzed with Wilcoxon signed ranks test and Spearman correlations. The level of statistical significance was defined as α = 0.05.

## Results

In table 1, descriptive statistics across the conditions are presented.

**Table 1:** Descriptive statistics for the slope across sound conditions for all variables.

**Variable**	**Sound**	**Mean**	**95% CI**	**Paired mean difference**	**95% CI**
**Mal** (Mal score/min)	Non-pos	1.80	1.14 to 2.46	0.27	-0.11 to 0.65
	Horizon	1.77	1.07 to 2.47		

**HR** (bpm/min)	Non-pos	0.79	0.41 to 1.17	0.00	-0.44 to 0.45
	Horizon	0.74	0.33 to 1.15		

**SCL** (μS/min)	Non-pos	0.76	0.46 to 1.07	0.17	-0.09 to 0.43
	Horizon	0.57	0.33 to 0.81		

**BVP** (a.u.^†^/min)	Non-pos	-0.10	-0.21 to 0.00	-0.09	-0.19 to 0.02
	Horizon	-0.02	-0.07 to 0.03		

**RR** (bpm/min)	Non-pos	-0.14	-0.29 to 0.00	-0.11	-0.26 to 0.03
	Horizon	-0.03	-0.15 to 0.10		

**Fixdur** (msec/min)	Non-pos	0.10	-0.64 to 0.84	0.47	-0.58 to 1.52
	Horizon	-0.26	-0.78 to 0.26		

**NoFix** (count/min)	Non-pos	2.45	0.79 to 4.11	2.16	-0.48 to 4.80
	Horizon	-0.02	-1.50 to1.45		

**Fixtime** (msec/min)	Non-pos	482.12	151.36 to 812.88	554.09	95.59 to 1012.58
	Horizon	-76.56	-327.59 to 174.47		

^†^Arbitrary units

### Perceived motion sickness

The average reported Mal score when the subjects terminated the trials was similar for both the sound horizon (22.9 points, SD 7.2) and the non-positioned sound condition (23.8 points, SD 6.2). Paired mean difference between the sounds was 1.1 points (95% CI -.8 to 3.9, p = 0.186) and the correlation was r = 0.67 (p = 0.001). Mal scores increased over time, i.e. Mal slope was significantly larger than zero (Table 1). The difference in Mal scores between the two sound conditions was not significant (Table 1).

### Duration of exposure

**Figure 2:**
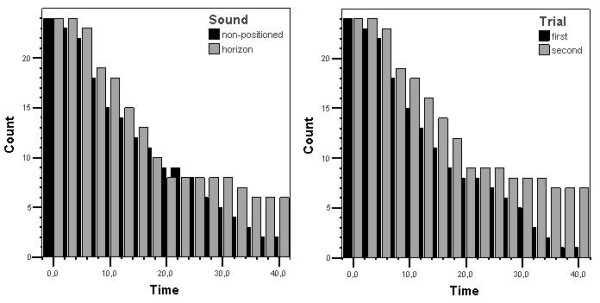
**Left table shows the number of subjects over time in the two sound conditions**. Right table shows number of subjects over time as a result of first and second trial.

Mean ST in the two sound conditions was 18.1 min (SD 12.3) for non-positioned sound and 20.2 min (SD 13.6) for the sound horizon, respectively (Figure 2). Paired samples t-test showed no significant difference in ST between the non-positioned sound and the sound horizon (p = 0.123).

However, a comparison of the first test trial versus the second test trial revealed a significant difference in ST (p < 0.001). Subjects endured the motion sickness stimulation longer when they came back for the second trial. Mean ST was 16.9 min (SD 11.7) for the first trial and 21.4 min (SD 13.8) for the second trial (Figure 2).

### Psychophysiological measurements

Temperature data were not normally distributed and therefore analyzed with non-parametric tests. Possible time effects, which is indicated by a non-zero mean slope, was investigated for non-positioned sound and sound horizon separately since measurements from the two sounds are not independent. HR and SCL slopes were significantly larger than zero (Table 1) indicating increase over time in both sound conditions whereas BVP and RR slopes were negative, however, not significant. Median temperature slopes were 0.004°C/min for non-positioned sound and 0.017°C/min for sound horizon. None of the psychophysiological variables had significantly different slopes in the non-positioned sound compared to sound horizon.

Pearson correlations were calculated between the slopes for all psychophysiological variables and the Mal slope. There was a significant positive correlation between Mal slope and HR slope as well as between Mal slope and SCL slope (Table 2).

**Table 2:** Correlations between Mal slope and slopes for the psycho-physiological and eye movement measurements.

	**HR**	**SCL**	**BVP**	**RR**	**Temp**	**Fixdur**	**NoFix**	**Fixtime**
**r**	0.59	0.61	0.29	0.16	0.14^†^	-0.11	0.45	0.38
**p**	< 0.001	< 0.001	0.058	> 0.3	> 0.3	> 0.3	0.003	0.013^††^

^† ^Spearman's rho

^†† ^Not significant after Bonferroni correction

### Eye movements

In the non-positioned sound, both Fixtime and NoFix increased over time, whereas none of them showed a significant time effect in the sound horizon (Table 1). Fixdur did not show a significant time effect in any of the sounds. Fixtime was the only variable showing significant differences between non-positioned sound and sound horizon. Fixtime slope was significantly larger in the non-positioned sound compared to the sound horizon (p = 0.02). Possible correlations between Fixtime, NoFix and Fixdur and Mal slope were checked for and a weak but significant positive correlation was found for NoFix, indicating that subjects reporting a large increase in Mal scores over time also increased their actual number of fixations (Table 2).

## Discussion

Mal scores increased over time in both sound conditions, but the artificial sound horizon, applied as a mitigation strategy for perceived motion sickness, showed no significant effect on Mal scores or ST. Based on these results, no effects of an artificial sound horizon as a mitigation strategy on perceived motion sickness could be identified. NoFix increased with time in the non-positioned sound condition. Moreover, fixation time increased faster in the non-positioned sound condition, on average with about half a second per minute, indicating that the subjects used more time to fixate and, hence, assumingly made fewer saccades [31]. This finding could be interpreted as a mitigation strategy applied by the subjects to cope with perceived motion sickness [16,17]. In the sound horizon condition, no such changes were found.

None of the other psychophysiological variables were affected by the artificial sound horizon. However, as Mal scores arose so did HR and SCL, indicating that these two variables are sensitive to motion sickness. In a previous study [32], HR turned out to be sensitive for motion sickness triggered in an optokinetic drum.

The results clearly advocate rejecting the tentative hypothesis that the artificial sound horizon could postpone the onset of perceived motion sickness or at least keep the symptoms at a constant lower level for some time, compared to the control condition. By scrutinizing the results further it can be concluded that the impact of the sound horizon was not large enough to be statistically significant with respect to how long the subjects endured the trials, i.e. ST, in the two different sound conditions. In the sound horizon condition, the subjects lasted on average 11% longer. The variation in Mal scores at the point of termination was large, but on average they were lower in the sound horizon condition; yet this finding was also not statistically significant. However, before rejecting the tentative hypothesis, the power of the study has to be in focus. Our within group design with 23 subjects did not yield a minimum required power of .80, given the ST and Mal scores outcome we have. Future research could thus replicate this study with a sufficient number of subjects, i.e. at least 3–5 times the number subject included in the present study, before a final decision on rejecting the tentative hypothesis should be made.

Using an artificial sound horizon means that the risk of adding conflicting cues – further triggering motion sickness development – will be considerably lowered, since vestibular and visual perception are the two dominating input channels triggering motion sickness. The idea behind the sound horizon is that it will work subliminally on the subject, which will lower the possible performance decline of the subjects when experiencing motion sickness. Sound could have a similar mitigating ability as the IVB used in the Duh et al. study [20], with the exception that it would not require any devoted cognitive attention. However, as shown in a study by Kennedy et al. [33] different visual patterns have different effect on perceived motion sickness. The same phenomenon is most likely to occur when using different sounds and auditory cues.

A confounding factor in the present study was revealed in the analyses of the impact of first versus second trial on ST. Previous experience obviously plays a crucial role [7-9]. Regardless which sound was presented, performing the trial the second time made the subjects endure 25% longer, a finding that, in fact, did reach statistical significance. Hence, based on these results it appears important to adjust the design in future research. A suggestion is to arrange the trials so that the subjects should get accustomed to become motion sick by a pilot trial carried out prior to the true trials, in order to lower the impact of first versus second experience of motion sickness.

Other factors that may have affected the outcome of the study were that the speaker positioning and the sound level of the two sounds could have been further improved. For example, the exact position of the speakers in terms of the sound spreading could have been optimized and measured. Carlander, Kindström, & Eriksson [25] have shown that the human ear can position sound sources with an exactness of 5° horizontally, but the accuracy vertically remains unknown. The sound horizon uses that vertical auditory positioning skill and a drawback of this study is that we do not know to which extent it was possible to detect the positioned sound vertically. Furthermore, the platform generated noise, and this noise may have interfered with the sound from the loudspeakers. Future experiments should thus try to minimize the influence of confounding sounds from the laboratory equipment. Since different sounds are perceived differently, future research should also investigate the effect on perceived motion sickness, using different kinds of sounds that are more naturalistic and thereby more subliminally effective. Furthermore, the assertion on having the sound horizon affecting the subjects subliminally could also have affected the results. If we had told the subjects about the sound horizon, it is possible that they in one instance could have perceived it as more helpful, but in another instance would have been forced to devote cognitive attention towards it. Since it is of great importance to collect subjective ratings of motion sickness as often as possible, it is always a trade-off between asking many or few questions to obtain a valid measurement of the perceived state.

The NoFix slope increased as Mal slope increased, a finding that was expected [16,17]. As mentioned, fixation time and NoFix are related measurements. Actually, fixation time is simply the multiplied product of NoFix and Fixdur, the latter showing no correlation with Mal scores. Furthermore, about a fifth of the Fixtime can be expected to have taken place while filling in the electronic questionnaire, further confounding the analyses of the eye tracking data. Hence, in the present study, clear cut conclusions from reduction of fixation time as a mitigation strategy for motion sickness among the subjects could not be drawn. However, as mentioned earlier Fixtime and NoFix increased over time in the non-positioned sound condition, indicating that eye movements seems to be sensitive to the artificial horizon.

In the present study, the variation in ST was large, and hence approximately half of the subjects terminated the tests before 50% of the maximum time had passed. Outcome data were analysed using a slope calculated as the termination minus baseline value divided by time. This approach assumes that subjects do develop motion sickness in a similar fashion but with different pace of development [34]. Adopting this approach allowed paired comparisons between the conditions regardless individual ST across conditions.

## Conclusion

A subliminally presented artificial sound horizon did not significantly affect perceived motion sickness, psychophysiological variables or the time the subjects endured the motion sickness triggering stimuli. The number of fixations and fixation times increased over time in the non-positioned sound condition, which was not the case in the sound horizon condition, indicating that eye movements could be a component of special interest to measure in future studies.

## Competing interests

The authors declare that they have no competing interests.

## Authors' contributions

JD carried out the planning, designing and the experimental trials. JD also participated in the analysis of the results and preparation of the manuscript. AS participated in the experimental trials, were responsible for the statistical analysis and participated in the drafting of the manuscript. TL participated in the drafting of the study and the final preparations before submission. TF Participated in the design and preparations of the study. TF also participated in the analysis and drafting of the manuscript. All authors have read and approved the manuscript.

## References

[B1] Cowings PS, Suter S, Toscano WB, Kamiya J, Naifeh K (1986). General autonomic components of motion sickness. Psychophysiology.

[B2] Crampton GH (1990). Motion and Space Sickness.

[B3] Hu S (1990). Motion Sickness Adaption: Changes in Psychological and Physiological Variables.

[B4] Cowings PS, Naifeh KH, Toscano WB (1990). The stability of individual patterns of autonomic responses to motion sickness stimulation. Aviat Space Environ Med.

[B5] Harm DL, Crampton GH (1990). Physiology of Motion Sickness Symptoms. Motion and Space Sickness.

[B6] Harm DL, Stanney KM (2002). Motion Sickness Neurophysiology, Physiological Correlates, and Treatment. Handbook of Virtual Environments.

[B7] Burcham PM (2002). Motion Sickess Literature Search.

[B8] Stern RM, Koch KL (1996). Motion Sickness and Differential Susceptibility. Curr Dir Psychol Sci.

[B9] Williamson JM, Thomas JM, Stern RM (2004). The contribution of expectations to motion sickness symptoms and gastric activity. J Psychosom Res.

[B10] Sugita N, Yoshizawa M, Abe K, Tanaka A, Watanabe T, Chiba S, Yambe T, Nitta S (2007). Evaluation of adaptation to visually induced motion sickness based on the maximum cross-correlation between pulse transmission time and heart rate. J Neuroeng Rehabil.

[B11] McClure JA, Fregly AR (1972). Forehead Sweating during Motion Sickness.

[B12] Murray JB (1997). Psychophysiological aspects of motion sickness. Percept Mot Skills.

[B13] Rolnick A, Gordon CR, Reuven G, Mangelsdorff DD (1991). The Effects of Motion Induced Sickness on Military Performance. Handbook of Military Psychology.

[B14] Lucot JB (1998). Pharmacology of motion sickness. J Vestib Res.

[B15] Magnusson M, Örnhagen H (1994). Rörelsesjuka- Sjösjuka. Översikt och utvecklingslinjer. FOA.

[B16] Flanagan MB, May JG, Dobie TG (2004). The role of vection, eye movements and postural instability in the etiology of motion sickness. J Vestib Res.

[B17] Webb NA, Griffin MJ (2002). Optokinetic stimuli: motion sickness, visual acuity, and eye movements. Aviat Space Environ Med.

[B18] Webb NA, Griffin MJ (2003). Eye movement, vection, and motion sickness with foveal and peripheral vision. Aviat Space Environ Med.

[B19] Bos JE, MacKinnon A, Patterson A (2005). Motion Sickness Symptoms in a Ship Motion Simulator: Effects of Inside, Outside and No View. Aviat Space Environ Med.

[B20] Duh HB-L, Parker DE, Furness TA (2001). An "Independent Visual Background" Reduces Balance Disurbance Evoked By Visual Scene Motion: Implication for Alleviating Simuator Sickness. SIGCHI; Seattle, WA USA.

[B21] Rolnick A, Bles W (1989). Performance and Well-being Under Tilting Conditions: The Effects of Visual Reference and Artificial Horizon. Aviat Space Environ Med.

[B22] Been-Lirn Duh H, Parker DM, Furness TA (2004). An Independent Visual Background Reducing Simulator Sickness in a Driving Simulator. Presence.

[B23] Petersen H, Magnusson M, Johansson R, Åkesson M, Fransson PA (1995). Acoustic cues and postural control. Scand J Rehabil Med.

[B24] Dozza M, Chiari L, Chan B, Rocchi L, Horak FB, Capello A (2005). Influence of a portable audio-biofeedback device on structural properties of postural sway. J Neuroeng Rehabil.

[B25] Carlander O, Kindstöm M, Eriksson L (2005). Intelligibility of stereo and 3D audio call signs for fire and rescue command operators. 11'th international conference of auditory display; Limerick, Ireland, July 5–9.

[B26] Graybiel A, Wood CD, Miller EF, Cramer DB (1968). Diagnostic Criteria for Grading the Severity of Acute Motion Sickness. Aerosp Med.

[B27] Miller EF, Graybiel A (1970). A Provocative Test for Grading Susceptibility to Motion Sickness, Yielding a Single Numerical Score Pensacola, Fla, USA.

[B28] Eye Tracking News, Head Mounted EyeTracker Systems. http://www.arringtonresearch.com/.

[B29] Falkmer T, Dahlman J, Dukic T, Bjällmark A, Larsson M (2007). Centroid vs. start point mode fixation identification in eye tracking data. Percept Mot Skills.

[B30] Altman DG (1991). Practical Statistics for Medical Research.

[B31] Yarbus AL (1967). Eye movements and vision.

[B32] Dahlman J, Sjörs A, Lindström J, Ledin T, Falkmer T (2008). Performance and autonomic responses during motion sickness. Hum Factors.

[B33] Kennedy RS, Stanney KM, Rolland J, Ordy MJ, Mead AP (2002). Motion sickness symptoms and perception of self motion from exposure to different wallpaper patterns. Human Factors And Ergonomics Society 46th Annual Meeting; September 30-October 4; Pittsburgh, PA.

[B34] Golding JF (2006). Motion Sickness Susceptibility. Auton Neurosci.

